# Multi-Omics Data Analysis Identifies Prognostic Biomarkers across Cancers

**DOI:** 10.3390/medsci11030044

**Published:** 2023-06-27

**Authors:** Ezgi Demir Karaman, Zerrin Işık

**Affiliations:** 1 Department of Computer Engineering, Institute of Natural and Applied Sciences, Dokuz Eylul University, Izmir 35390, Turkey; ezgi.demir@ceng.deu.edu.tr; 2 Department of Computer Engineering, Faculty of Engineering, Dokuz Eylul University, Izmir 35390, Turkey

**Keywords:** multi-omics data, network-based integration, community detection, survival analysis, cancer biomarker

## Abstract

Combining omics data from different layers using integrative methods provides a better understanding of the biology of a complex disease such as cancer. The discovery of biomarkers related to cancer development or prognosis helps to find more effective treatment options. This study integrates multi-omics data of different cancer types with a network-based approach to explore common gene modules among different tumors by running community detection methods on the integrated network. The common modules were evaluated by several biological metrics adapted to cancer. Then, a new prognostic scoring method was developed by weighting mRNA expression, methylation, and mutation status of genes. The survival analysis pointed out statistically significant results for *GNG11*, *CBX2*, *CDKN3*, *ARHGEF10*, *CLN8*, *SEC61G* and *PTDSS1* genes. The literature search reveals that the identified biomarkers are associated with the same or different types of cancers. Our method does not only identify known cancer-specific biomarker genes, but also proposes new potential biomarkers. Thus, this study provides a rationale for identifying new gene targets and expanding treatment options across cancer types.

## 1. Introduction

Cancer is a heterogeneous disease caused by changes in cell behavior, uncontrolled growth and genomic alterations such as mutations. It contains many different forms, variables and multiple subgroups. In 2020, a total of 19.3 million new cancer cases occurred in the world and there were almost 10 million cancer-related deaths [1]. The most diagnosed cancers were breast (11.7%), lung (11.4%) and colorectal (10%), while cancer-related deaths occurred most oftenwith lung (18%), colorectal (9.4%), liver (8.3%), stomach (7.7%), and breast (6.9%) cancers [1]. If the incidence rates continue at the same frequencies, it is estimated that there may be 28.4 million new cancer cases in 2040 [1]. For a better prognosis and treatment process in such a disease, it is important to categorize tumors into genetically similar subgroups and associate these subgroups with clinical outcomes. Identifying key genomic similarities shared between cancer types will allow extending effective treatments in one cancer type to others due to sharing similar genomic profiles [2].

The complex biology of cancer diseases cannot be explained by analyzing a single omic data type. A wealth of omics data from genomes, transcriptomes, proteomes, metabolomics, ionomics and epigenomes provide a comprehensive perspective for researchers to better explore cancer biology [3]. The availability of such data requires integrative methods to make further evaluations. The use of cancer informatics methods, which integrate and interpret genome-scale molecular data, may reveal possible biomarkers related to tumor prognosis, diagnosis, etc. For this purpose, various clustering algorithms and advanced analysis techniques can be applied to integrated data [4].

In recent years, biological networks, as a simple but effective representation of complex interactions and regulatory relationships between molecules, have been used extensively to understand the system-level characteristics of diseases [5]. Integrating different types of omics data on these networks and applying network-clustering methods to the integrated data may give more effective results inrevealing biomarkers of cancer development or prognosis [6].

Multi-omics data integration methods can be grouped as deep learning networks, network-based, clustering, features extraction, transformation and factorization [7]. These methods address various applications such as disease subtyping, biomarker discovery, pathways analysis and drug repurposing [7,8].

Here, we focus on specific studies that integrate different types of omics data using network-based approaches and use them for biomarker discovery. Kim et al. [9] proposed a random walk approach on an integrated gene–gene graph with expression and methylation profiles; their analysis identified cancer-specific pathways covering genes related to breast cancer. Another research identified differentially expressed and methylated genes and miRNAs for lung adenocarcinoma, integrated the common genes into the PPI network structure and determined potential target genes as a result of survival analysis [10]. There are some studies that aim to find diagnostic and prognostic biomarkers in endometrial, prostate, and colorectal cancers by applying a similar approach using DNA methylation and gene expression data [11,12,13]. Sun et al. [14] performed an integrated analysis of genome-wide DNA methylation and gene expression for hepatocellular carcinoma, applied a weighted gene co-expression network analysis (WGCNA) and survival analysis; and found gene signatures associated with overall survival. Champion et al. [15] developed a new algorithm and identified potential cancer drivers for eleven cancer types, including breast invasive carcinoma (BRCA), colorectal adenocarcinoma (COAD), lung squamous-cell carcinoma (LUSC), and kidney renal clear-cell carcinoma (KIRC), with the integration of copy number, DNA methylation and gene expression data. Dimitrakopoulos et al. [16] proposed a network-based integration of the multi-omics data (NetICS) method to prioritize cancer genes. The SNF method [17], which was also applied in this study to integrate gene expression and DNA methylation data, is one of the commonly used methods developed for subgroup identification. Furthermore, it has also been applied to prioritize candidate disease genes [18] and to identify candidate cancer biomarkers [19].

There are studies that apply community detection methods instead of traditional clustering algorithms for cancer biomarker discovery. Tanvir and Mondal [20] determined possible biomarkers for COAD, BRCA and glioblastoma multiforme (GBM) by running seven community detection algorithms on gene co-expression networks. Another study applied a community detection algorithm to a differential gene regulatory network created for breast cancer and suggested diagnostic biomarkers [21]. Yu et al. [22] applied the MCODE algorithm to co-expression networks of multiple cancers to find biomarkers. However, these studies only consider gene expression data rather than an integrated multi-omics network. In addition, while evaluating clustering algorithm performances in most studies, statistical metrics were used instead of biological metrics. Despite that, the extent to which genes in the same module are biologically homogeneous is important for biomarker discovery.

Although different types of omics data were used as biomarkers, the expression data of several genes wereused as a scoring value in the survival analysis [23,24,25]. To the best of our knowledge, there is no integrative scoring method which concurrently combines different omics data for performing the survival analysis.

In this study, different types of omics data of lung, breast, colorectal and kidney cancers, which are at the forefront in terms of both mortality and incidence, were analyzed. RNA-sequencing and DNA methylation data are integrated into a network. Various network clustering algorithms were applied to the integrated data. Biological metrics were used to evaluate clustering results. For this purpose, a metric called “bioscore” has been developed that examines only cancer-specific biological functions and pathways in clustering evaluation. The same analysis workflow was applied to the validation set and prospective biomarkers were selected. In addition, the mutation status of these biomarker genes was also investigated. Finally, survival analysis was conducted with a new prognostic scoring method developed by using different omics data. The obtained biomarkers were compared with studies in the literature. Some studies present these genes as biomarkers for lung, colorectal, breast and kidney cancers, in line with our study. On the other hand, there are other studies suggesting some genes as biomarkers for other cancer types such as prostate, gastric, hepatocellular, ovarian, and bladder. From this point of view, our study helps to reveal genomic similarities among various cancer types. Moreover, some potential novel biomarkers have been found that need to be confirmed by further wet-lab studies.

## 2. Materials and Methods

The data set and stages of the method are presented in this section. Gene expression, DNA methylation and somatic mutation data of four cancer types (BRCA, KIRC, LUSC, COAD) were obtained from the publicly available TCGA projects [26]. The dataset was divided into two parts for using different patient samples in training and validation of the model.

**Figure 1:**
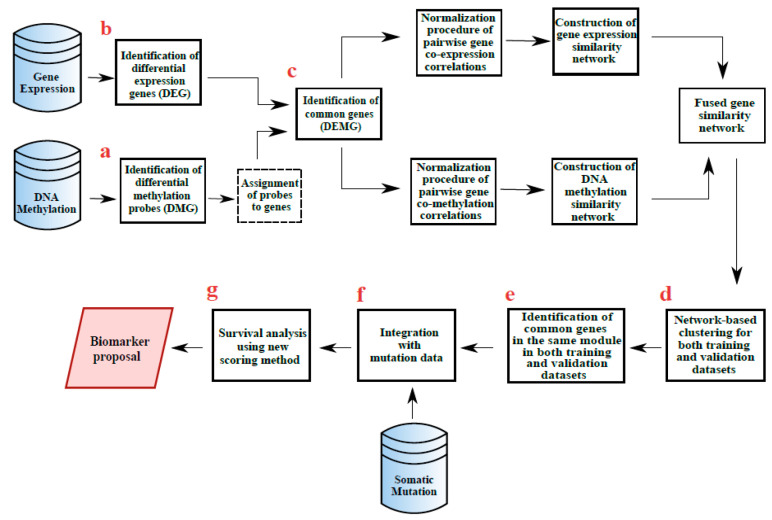
Overview of the study workflow. (**a**) Differentially methylated genes (DMG-hypo/hyper) were obtained. (**b**) Differentially expressed genes (DEG) were obtained. (**c**) Differentially expressed and differentially methylated genes (DEMG-hypo/hyper) were obtained by taking the common ones between these two groups. (**d**) Clustering algorithms were implemented to detect modules on the DEMG-hyper and DEMG-hypo networks for both training and validation sets. (**e**) Common genes of all cancer types and that were included in the same modules in the training and validation datasets were determined. (**f**) The mutation status of each gene was examined. (**g**) The potential biomarker genes were identified through survival analysis based on the developed prognostic scoring method.

Figure 1 provides an overview of the methods used in the study. First, differentially expressed genes and methylated probes were identified. Then, probes with significant methylation changes were paired with the 10 closest upstream and downstream target genes with significant expression changes. Using these probe-gene pairs, the mean value of the probes was assigned to each gene. After that, common differentially expressed and methylated genes were identified. Co-expression and co-methylation networks were constructed with these genes. Co-expression and co-methylation networks were integrated by Similarity Network Fusion (SNF) [17]. Network clustering algorithms run on the resulting integrated networks for each cancer type. Clustering results were evaluated by using biological metrics and the most biologically significant modules were determined. The same pre-processing and analysis methods were applied to both the training and the validation data sets. Common genes (i.e., biomarkers) identified in the same module for both training and validation datasets in four cancer types were extracted. The mutation status of each biomarker gene was examined and the genes covering most mutations for all cancer types were determined. In addition, survival time analysis was applied to observe the effects of biomarker genes; eventually, a scoring method was proposed for survival forecasting.

### 2.1. Data Analysis

We retrieved DNA methylation, gene expression and somatic mutation data for four different cancer types available on the TCGA website: COAD, KIRC, BRCA and LUSC [26]. We selected these tumors based on analysis of the Pan-Cancer Project [2], which focused on 12 tumor types. Due to the higher number patient samples for three omics data and literature comparability, we focused on four of them (COAD, KIRC, BRCA, LUSC). The TCGAbiolinks package was used to retrieve TCGA data from the GDC data portal [27]. Then, patients having both gene expression and DNA methylation data types were determined. Data from untreated patients were used because some treatments may cause changes in omics data. To avoid misleading results, untreated patients in stage-I and stage-II were filtered out. The data were divided into two sets, training and validation, by random split. Table 1 shows the number of samples for both training and validation datasets. In addition, baseline clinical characteristics were presented in the File S1. The Chi-Square test was used to compare the differences in clinical variables between the training and validation data sets.

#### 2.1.1. Identification of Differentially Expressed Genes

Differential gene expression analysis was performed to identify gene expression changes between the tumor and normal samples. In this analysis, the edgeR package is used and both exacttest and log2 foldchange were calculated. The *p*-values were adjusted using Benjamini and Hochberg’s approach [28]. Statistically significant gene lists were obtained by filtering genes with the absolute log2 foldchange value > 1.0 and FDR < 0.05.

#### 2.1.2. Identification of Differentially Methylated Probes

We aimed to identify DNA methylation changes in distal regulatory regions and correlate these signatures with mRNA expression in nearby genes. Identification of differentially methylated probes, binding of distal probes with significant methylation changes to target genes, and selection of probe-gene pairs were performed by using the ELMER package [29].

ELMER analysis [29] uses a data structure called “MultiAssayExperiment” (MAE) which stores different assays of all samples in a single object. A “MAE” object containing DNA methylation and gene expression data was created using the “createMAE” function. Using the “get.feature.probe” function provided by ELMER, only distal probes (at least 2 Kbp away from the transcription start site) were selected; thus, we aimed to identify distant interactions that regulate genes. In this function, the “genome” parameter is set to hg38, and the “met.platform” parameter is set to 450 K. The determined distal probes were given as the “filter.probe” parameter of the “createMAE” function. After this step, differentially methylated CpGs were identified using the “get.diff.meth” function, which performed a one-way *t*-test. The “sig.dif” parameter of this function, which indicates the smallest DNA methylation difference, is a cutoff value for selecting significant hypo-/hyper-methylated probes and it was set to 0.3. Since the group structure (tumor vs. normal) in the analysis was known in advance, the “mode” parameter was chosen as supervised. Raw *p*-values were adjusted by using the Benjamini–Hochberg method [28], and probes with adjusted *p*-value < 0.01 were selected. The next step of the analysis is to identify probe–gene pairs. Using the “get.pair”function, selected distal probes with significant methylation changes were linked to the closest 10 upstream and 10 downstream target genes with significant expression changes. Silva et al. [29] aimed to avoid systematic false positives for probes in gene-rich regions by choosing a fixed number of genes to be tested for each probe. In this function, the “filter.percentage” and “filter.portion” parameters are set to 0.05 and 0.3, respectively. This setup guarantees that at least 5% of beta values are less than 0.3 and 5% of beta values are greater than 0.3.

### 2.2. Construction Gene Co-Expression & Co-Methylation Networks

Using the probe–gene pairs determined in the previous step, the average methylation value of the probes was assigned to each gene. The Ensemble gene identifiers were converted to the Entrez gene identifiers by using the “org.Hs.eg.db” package [30]. Then, common differentially expressed-hypomethylated genes (DEMG_Hypo), and differentially expressed-hypermethylated genes (DEMG_Hyper) were identified.

While constructing a co-expression and co-methylation network, we used these common genes specific to each cancer type. A correlation value between two genes is computed by the normalized absolute Pearson correlation with the same method as given in a previous study [31]. First, the expression and methylation correlation coefficients between two genes were computed using Pearson correlation. The Fisher transform was applied to make comparable correlation estimates between datasets. We standardized values as *z*-scores in each dataset. Then, the standardized correlations were obtained by inverting the *z*-score. The absolute value of correlations is used as the edge weight of both co-expression and co-methylation networks. The algorithm is summarized in the ‘Algorithm 1’ section below. This method was applied to all types of cancers (i.e., BRCA, COAD, KIRC, LUSC).
**Algorithm 1: Procedure for determining pairwise gene correlations.****Input:** expression and methylation profiles of *n* genes.**Output:** pairwise gene correlations *r^′^_ij_* for any pair of genes *i* and *j*.**Compute correlation** *r_ij_* of each pair of genes *i* and *j*, using Pearson correlation.**Normalize** *r_ij_* for any 1 ≤ *i*, *j* ≤ *n* with the following steps:**1.** Apply Fisher’s *z* transformation to *r_ij_*, i.e., $=0.5ln\left(\frac{1+r_{ij}}{1-r_{ij}}\right)$*z_ij_***2.** Standardize *z_ij_*, i.e., *z^′^_ij_*= $\frac{z_{ij}-\mu }{\sigma }$, where μ and σ are the mean and standard deviation of *z_ij_* for all 1 ≤ *i*, *j* ≤ *n.***3.** Apply Fisher’s inverse transformation to *z^′^_ij_*, i.e., *r^′^_ij_*=$\frac{exp(2{z’}_{ij})-1}{\exp\left(2{z’}_{ij}\right)+1}$ **Return** *r^′^_ij_* for any *i*, *j*.

### 2.3. Network-Based Data Integration

Co-expression and co-methylation networks individually created for each cancer type were used as the input of an integrative method called Similarity Network Fusion (SNF) to construct a weighted and undirected similarity network [17].

SNF is based on a certain number of similarity matrices corresponding to different layers referring to the same set of nodes. The similarity matrices are then converted into a unique similarity matrix. During this transformation, SNF has the purpose of strengthening the weaker links common to all layers as well as the very strong links found in one layer. The nodes of the obtained network are the common ones in each layer, and the edges are calculated according to the new similarity values. There are three parameters in SNF: *K* is the number of neighbors, *α* is a hyper-parameter, and *t* is the number of iterations. We ran the SNF algorithm with the *K* value as 5, 9, 21, and 30 and the *t* value as 5, 10, and 20. However, we obtainedmore stable results by setting *K* = 9 and *t* = 20. This setup was used for all cancer types.

After the *t* steps of iteration, co-expression and co-methylation networks converge to integrated gene similarity networks. We used a min-max normalization for these networks to obtain more stable results. The adjacency matrix obtained as a result of SNF was converted into a graph using the “igraph” package [32].

### 2.4. Network-Based Clustering

Fast Greedy [33], Infomap [34] and Louvain [35] clustering algorithms run on integrated gene similarity networks specific to each cancer type. Fast Greedy tries to find communities in graphs by optimizing the modularity score, which is based on the idea of having dense connections between nodes within modules but having sparse connections between nodes of different modules [33]. Infomap finds a community structure that minimizes the expected description length of a random walker trajectory [34]. Louvain implements the multi-level modularity optimization algorithm for finding a community structure. It is based on the modularity measure and a hierarchical approach [35]. Each clustering algorithm runs using the corresponding functions of the igraph library with its default parameters [32].

BHI and Bioscore metrics were used for the evaluation of the clustering results. The BHI measures how biologically homogeneous the clusters are [36]. The measure checks whether genes found in the same cluster also belong to the same biological function classes. The BHI is in the range of [0,1]; larger values correspond to more biologically homogeneous clusters. The “BHI” function in the “clValid” library was used to calculate the BHI score.

Another biological metric is the Bioscore, which was adapted based on the work of Bruno and Friori [37]. According to their work, this score assessed how many gene subsets showed a significant *p*-value considering all function classes. However, there were many functional terms that are unrelated to cancer development. Therefore, we adapted the Bioscore metric to measure the homogeneity of clusters by scoring only the cancer-related Gene Ontology (GO) Biological Processes (BP) and KEGG pathway terms. The cancer-related GO BP and KEGG pathway terms are taken from the study of [38]. Fisher’sexact test [39] was used to identify significant terms and raw *p* values were adjusted using the Benjamini–Hochberg method [28] and terms with adjusted *p* < 0.05 were considered significant. If a gene in a cluster is involved in a significant cancer-related GO BP or KEGG pathway, the score of this gene increases by 1, otherwise it remains 0. After calculating a score for each gene in a cluster, they are summed, and a min-max normalization is applied to ensure consistency across all clusters. The Bioscore of a cluster is

(1) $$Bioscore=\sum_{i}^{K}\sum_{cat}^{G}\Theta_{i,cat}$$

where *K* is the number of genes in the dataset, and *G* is the number of cancer-related and functional categories stored in the external file. These cancer-related terms are given in Table S1 for GO BP and in Table S2 for the KEGG pathway. *Θ_i,cat_* is defined as follows:

(2) $$\Theta_{i,cat}=\left\{\begin{array}{c} 1,P_{i,cat}<t\\ 0,otherwise \end{array}\right.$$

where *P_i,cat_* is the *p*-value of the cancer-related category *cat* associated with gene *i*, and *t* is a threshold (e.g., 0.05). The most biologically homogeneous modules were determined by examining the results obtained.

### 2.5. Validation Analysis

The same pre-processing and analysis methods were applied to the validation samples that are given in Table 1. Statistically significant modules were obtained by applying clustering to the validation dataset. Common genes, which are found in the same module for both training and validation datasets, were identified for all cancer types. Then, these genes were selected for biomarker analysis.

### 2.6. Somatic Mutation Status of Biomarkers

Somatic mutation data of BRCA, LUSC, KIRC, and COAD cohorts were downloaded from the GDC Portal. The mutation data were filtered based on biomarker genes identified in the previous step for untreated patients in stageI and stage II. The mutation status of each biomarker was examined and the genes with the highest number of mutations were determined for all cancer types.

### 2.7. Survival Analysis

After identifying biomarker genes, the effects of these genes on the overall survival time of patients were also investigated. For this purpose, a new scoring scheme was created by taking a weighted summation of individual scores of DNA methylation, gene expression and mutation data. We called this score “prognostic score”, since this score would show both positive (e.g., high prognostic score → good survival) and negative (e.g., high prognostic score → poor survival) correlation with the survival time of a patient.

The prognostic score by considering three data types is calculated by the following equation:

(3) $$Prognosticscore\left(g_{x}\right)=Geneexpression\left(g_{x}\right)\times 0.5+DNAmethylation\left(g_{x}\right)\times 0.3+Mutationstatus\left(g_{x}\right)\times 0.2$$

where *g_x_* represents a gene. For this procedure, a log transformation followed by a min-max normalization was applied to the raw read counts of RNA-sequencing. Mutation status was assigned “1” if the gene has a mutation, otherwise “0”. Since the beta value varies between 0 and 1 in DNA methylation, it remains the same value. For survival analysis, continuous values should be represented as categorical values. For this process, the differentially expressed and hypomethylated genes (DEMG_Hypo) and differentially expressed and hypermethylated genes (DEMG_Hyper) were compared among themselves by cancer type. Consequently, common DEMG Hypo and common DEMG Hyper genes were identified in both the training and validation sets for each cancer type. The numbers of these genes, named DEMG_Common, are shown in Table 2.

The prognostic score value was calculated for all DEMG_Common given in Table 2.

(4) $$HighLevelforg_{x}=\frac{\sum Prognosticscore(g_{x})}{Numberofpatientsincancertype}+SD(Prognosticscore(g_{x}))$$

(5) $$LowLevelforg_{x}=\frac{\sum Prognosticscore(g_{x})}{Numberofpatientsincancertype}-SD(Prognosticscore(g_{x}))$$

where *g_x_* represents a gene. High and low levels were determined by taking the mean +/− 1-standard deviation of each gene’s score for all patients (Equations (4) and (5)). After calculating these values for all genes, the average high and low cutoff values were obtained by dividing by the number of DEMG_Common in each cancer type *T* (Equations (6) and (7)).

(6) $$Avg.ofHighLevels(T)=\frac{\sum HighLevelforallg_{x}}{NumberofDEMG_{Common}incancerT}$$

(7) $$Avg.ofLowLevels(T)=\frac{\sum LowLevelforallg_{x}}{NumberofDEMG_{Common}incancerT}$$

According to these limits, a score of a gene less than average low level was labeled as “low”, one between average low and high level as was labeled “normal”, and one higher than average high level as was labeled “high”. Finally, we obtained a categorical prognostic score for each gene.

The Cox proportional hazard model and “survival” package were used to analyze the risk factors [40]. To perform survival analysis, vital status, days to last follow-up and days to death information were obtained from the clinical data files of the patients. The time variable was taken as the days to the last follow-up if the patient was alive, and as the days to death if the patient was dead. In addition, to understand the relationship between categorical variables and overall survival, the Kaplan–Meier estimator [41] was used, which is one of the most widely-used non-parametric measures in survival analysis and in medical research.

Another point we would like to mention is that gene expression (0.5), DNA methylation (0.3), and mutation (0.2) weights are not arbitrarily selected in the prognostic score equation. We also experimentally tested the version of the weights with gene expression (0.4), DNA methylation (0.4) and mutation (0.2). However, in the analysis carried out with this version (0.4, 0.4, 0.2), we obtained fewer biomarkers based on significant hazard ratio and *p*-values. Considering that there are no mutation data for each gene, we assigned the smallest weight (0.2) to the mutation data in both versions. Since survival analysis studies are mostly based on gene expression, we decided to use the weight combination to place more emphasis on gene expression.

Moreover, in order to evaluate the power of survival analysis by combining the three data types, we also computed the prognostic score (Equation (3)) by using a single data type (gene expression, DNA methylation, or mutation status). For this process, the same pipeline described above was applied to each data type. For gene expression and DNA methylation, high and low cutoff values were determined independently, and survival analysis was carried out by labeling in accordance with these cutoffs. Since the mutation status is represented as binary data (value of “1” indicates mutation, otherwise it becomes “0”), survival analysis with mutation status was performed by directly using these binary values.

### 2.8. MOFA Analysis

We applied the Multi-Omics Factor Analysis (MOFA), which is a computational method used to gain biological insights from multi-omics data. SNF combines multi-omics data through network fusion, whereas MOFA applies a matrix factorization for data integration. MOFA is an adaptation of Principal Component Analysis (PCA) for multi-omics data. MOFA takes data matrices from each omics type as input, and then decomposes these matrices into a factor matrix for each sample and weight matrices for each omics data type [42].

The same samples (given in Table 1) and the three omics layers of DEMG_Common (mentioned in Table 2), gene expression, DNA methylation, and somatic mutation were used in the MOFA implementation. In addition, information from patients’ clinical data files was also included as metadata. For the gene expression data, a log transformation followed by a min-max normalization was applied to the raw read counts. Mutation status was assigned “1” if the gene has a mutation, otherwise “0”. Since the beta value ranges between 0 and 1 in DNA methylation, it remains the same value. After data preprocessing, we used the R package MOFA [42], an unsupervised factor analysis model to perform multi-omics data integration. We employed default parameters for model training (number of factors = 15, convergence mode = “slow”, maxiter = “1000”, seed = “42”).

Next, we aimed to understand the molecular etiology of the MOFA factors. We investigated whether any of the inferred latent factors were related to prediction of patient outcomes by using the Cox proportional hazards model. Evaluating top weights using the loadings of each feature can provide us with insights for identifying clinical biomarkers. Therefore, across all omics data types, we selected the top 30 genes with the highest weights in the significant factors identified through survival analysis. In addition, for each omics data type, we identified the 30 highest weighted genes in the first three components that were shown to be significant as a result of the variance decomposition analysis performed with MOFA. We examined the associations of these genes with the previously identified potential biomarkers.

## 3. Results

The results of the entire analysis are summarized in this section.

### 3.1. Identification of Differentially Expressed Genes/Differentially Methylated Probes

Table 3 showsthe number of significant hypo-/hyper-methylated probes, the number of 10 closest upstream and 10 downstream target genes to probes with significant methylation changes, and the number of statistically significant ones among these probe-gene pairs for the training set. The same analysis was also applied for the validation set and the statistics are given in Table 4.

For the probe-gene pairs determined in the previous step, the mean methylation values ofprobes matching a gene were assigned this gene. Table 5 summarizesthe number of differentially methylated genes obtained in this way (DMG-hypo/hyper) (Figure 1a), the number of differentially expressed genes (DEG) (Figure 1b) and the number of both differentially expressed and differentially methylated genes (DEMG-hypo/hyper) (Figure 1c) obtained by taking the common ones in these two groups for the training set. The same analysis was also applied for the validation set and the same statistics are given in Table 6. The next analysis steps were continued with the genes in the DEMG-hypo/hyper group.

### 3.2. Identification of Common Genes in Different Cancer Types

**Figure 2:**
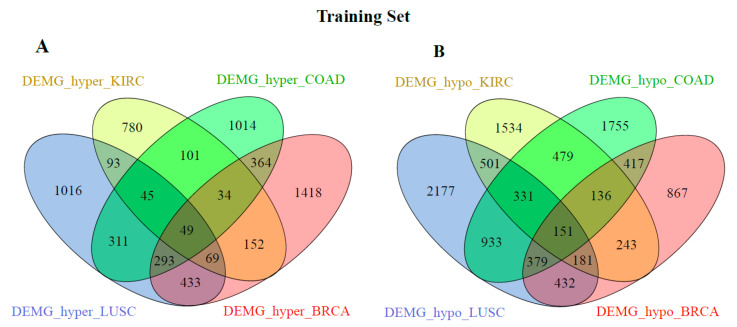
Distribution of training samples (**A**) DEMG_hyper and (**B**) DEMG_hypo between cancer types without applying SNF and clustering algorithms.

**Figure 3:**
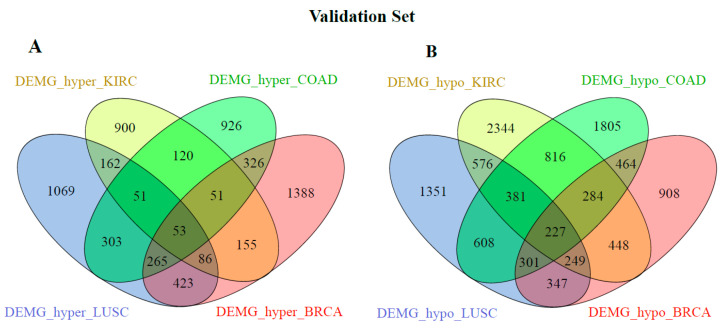
Distribution of validation samples (**A**) DEMG_hyper and (**B**) DEMG_hypo between cancer types without applying SNF and clustering algorithms.

Figure 2 and Figure 3 show the distribution of the DEMG_hyper and DEMG_hypo for training and validation data before applying SNF and clustering algorithms. As seen in these figures, 49 DEMG-hyper and 151 DEMG_hypo genes were found for the training set, and 53 DEMG-hyper and 227 DEMG_hypo common genes were found for the validation set.

**Figure 4:**
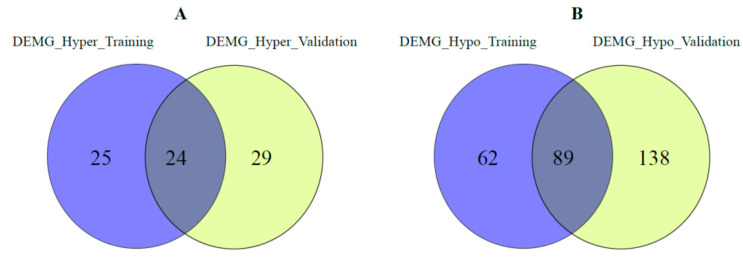
Comparison of (**A**) DEMG_hyper genes common to all four cancer types for validation and training sets, (**B**) DEMG_hypo genes common to all four cancer types for validation and training sets.

In addition, for the training and validation set, we compared the DEMG_hyper and DEMG_hypo genes common to all four cancer types among themselves. The distribution of these genes is given in Figure 4. Most of the common genes were found in both the training and validation sets.

### 3.3. Network Clustering

The biologically homogeneous modules offour cancer types were compared to reveal potential common biomarker genes related to these cancers. For this purpose, we have implemented Fast Greedy, Infomap and Louvain clustering algorithms to detect modules on the DEMG-hyper and DEMG-hypo networks for both training and validation sets (Figure 1d). The performance of each algorithm was evaluated by using both BHI and Bioscore metrics; these results are summarized in Table 7, Table 8, Table 9 and Table 10. As seen in Table 7 and Table 8, the Fast Greedy algorithm gave higher BHI and Bioscore for all DEMG-Hyper and DEMG-Hypo data for the training set. As seen in Table 9, fast greedy algorithm for BRCA and COAD, Louvin algorithm for LUSC and KIRC gave the best results for DEMG_Hyper data for the validation set. As seen in Table 10, Fast Greedy algorithm for BRCA, COAD and KIRC and Louvin algorithm for LUSC gave the best results for all DEMG_Hypo data for the validation set.

The modules obtained by the clustering algorithm, which gave a better result for each cancer type, were compared among themselves as the DEMG_Hyper ones and the DEMG_Hypo ones. Then, genes that are common to all cancer types and that were included in the same modules in the training and validation datasets were determined (Figure 1e). These genes are listed in Table 11.

### 3.4. Somatic Mutation Status of Biomarkers

**Figure 5:**
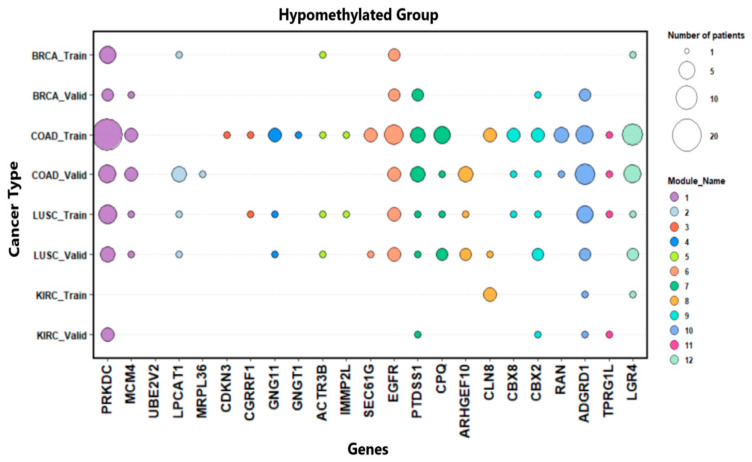
Distribution of patients with mutated genes in the hypomethylated group by cancer types.

The mutation status of each gene in Table 11 was also examined (Figure 1f). Figure 5 shows the number of patients with gene mutations for the hypomethylated group. The color of the bubbles was used to represent the genes in the same modules, and bubble size represents the number of patients. For this procedure, we normalized the number of patients with mutations in that gene by the total number of patients with mutations in each cancer type. The genes having the most mutations for the hypomethylated group were PRKDC, EGFR, PTDSS1, ADGRD1 and LGR4, while SLC9A3 and BRIP1 were the most mutated ones for the hypermethylated group.

**Figure 6:**
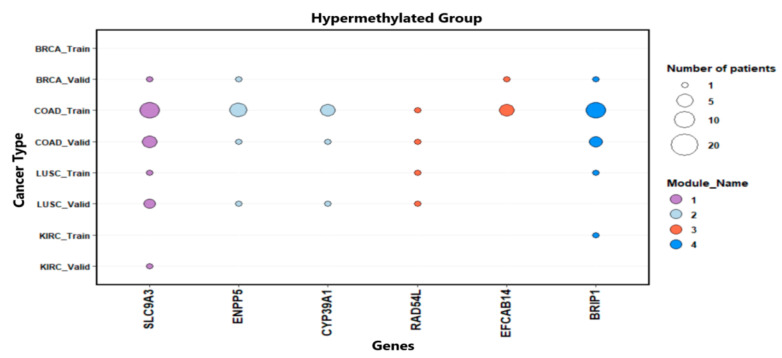
Distribution of patients with mutations of genes in the hypermethylated group by cancer types.

Figure 6 shows the number of patients with gene mutations for the hypermethylated group. It was observed that the mutations were mostly of the “missense” type for both groups.

### 3.5. Survival Analysis

Survival analysis was performed for the genes given in Table 11. The “prognostic score” described in Section 2.7 was used for this analysis. Since this is a continuous value, it must be converted into a categorical value for survival analysis. Therefore, high and low limits were determined by taking the mean +/− 1-standard deviation of each gene’s score for all patients. After calculating these averages for all genes, high- and low-level cutoffs were determined based on the average of high- and low-level scores computed specifically for each cancer type. These values were summarized in Table 12. According to these limits, a score less than average low level was labeled as “low”, one between average low and high level was labelled as “normal”, and one higher than average high level was labelled as “high” for each cancer type.

The results of the survival analysis with hazard ratio > 1.0 and *p*-value < 0.05 are presented in Table 13. Among the significant results are potential biomarker genes that were determined by considering the number of patients at that level and the number of deaths according to the prognostic score (Figure 1g). In addition, Kaplan–Meier plots of these genes are presented in File S2.

We additionally tested the weights with gene expression (0.4), DNA methylation (0.4), and mutation (0.2). Based on a significant hazard ratio and *p*-values, we found fewer biomarkers in the analysis carried out with this version (0.4, 0.4, 0.2). We present the proof of this analysis result in File S3.

### 3.6. Usage of Individual Data Types for Survival Analysis

Another survival analysis was performed by using gene expression, DNA methylation and mutation data individually to compare them with the proposed multi-omics prognostic score. The survival analysis based on individual data types presented fewer significant results when the hazard ratio and *p*-values were considered. The File S4 summarizes the results of survival analysis with individual data types. We claim that the new prognostic scoring by integrating multi-omics data would empower common biomarker identification across tumor types.

### 3.7. MOFA Analysis

MOFA was applied to gene expression, DNA methylation and somatic mutation data of four cancer types (BRCA, COAD, KIRC, and LUSC). Significant factors in the trained models were used in the survival analysis. From the identified 15 MOFA factors, Factor 7 (*p*-value = 0.0001), and Factor 15 (*p*-value = 0.0198) for BRCA_hypo, Factor 7 (*p*-value = 0.0201) for COAD_hypo, Factor 8 (*p*-value = 0.0005) for KIRC_hypo, Factor 12 (*p*-value = 0.016) for LUSC_hypo, and Factor 4 (*p*-value = 0.04) and Factor 8 (*p*-value = 0.015) for BRCA_hyper were statistically significantly associated with overall survival. We identified the top 30 genes with the highest weight in these factors and the first three factors determined by variance decomposition analysis. We observed some similarities between these genes and the results provided in Table 11 and Table 13. For example, the survival analysis of two methods identified CBX2 and GNG11 genes in BRCA_hypo phenotype. Further results are presented in File S5.

## 4. Discussion

In the first stage of the study, we performed a network-based integrative analysis with the SNF method using DNA methylation and gene expression data of BRCA, COAD, KIRC and LUSC. Community detection methods were applied to the integrated network and the results were evaluated using cancer-related biological metrics. The same procedure was implemented on both training and validation datasets for all cancer types. As a result of this procedure, there is a concordance between the genes identified in the same module for both training and validation data sets (Table 11). Some of these genes were also mentioned in previous studies in the literature. These studies integrated various omics profiles (e.g., gene expression, DNA methylation, somatic mutation, copy number) and applied one or more computational approaches such as a deep neural network, co-expression network, feature selection, differential expression or methylation gene analysis, or protein–protein interaction analysis on different cancer types. For instance, Fan et al. [43] identified triple-evidence genes representing differentially methylated, differentially expressed, and somatic mutation-associated genes in each of the 13 TCGA cancers. Among the triple-evidence genes they determined, the genes that were also common to all four cancer types in our study are as follows: CBX2, CBX8 genes for BRCA, LUSC and COAD; MCM4, GNG11 genes for LUAD; LGR4 gene for COAD, KIRC, LUSC; LPCAT1 gene for LUSC; EGFR gene for KIRC and ARHGEF10 gene for BRCA. In another study, Mo et al. [44] performed a statistical integrative clustering analysis (iCluster+) using exome sequence, DNA copy number, promoter methylation, and mRNA expression data of TCGA colorectal carcinoma. In this analysis to discover cancer subgroups, PTDSS1, MCM4 and PRKDC genes were identified as molecular drivers belonging to the same subgroup. Qi et al. [45] constructed a PPI network with differentially expressed and aberrantly methylated genes for breast cancer and identified MCM4, CDKN3 and EGFR as hub genes. In another breast cancer study using gene expression and copy-number alterations data in a neural network-based approach, the CDKN3 was one of the subtype-specific genes identified belonging to the LumA subtype [46]. Fiorentino et al. [47] developed a methodology that fuses omics-specific similarity networks in a single network and verified the SEC61G gene as a prognostic biomarker using gene expression, methylation, and miRNA data of GBM. Dimitrakopoulos et al. [16] identified the known EGFR gene for lung cancer by their proposed network-based integration method using somatic mutations, copy number variations, methylation, mRNA and miRNA expression data. Sheng et al. [48] identified differentially expressed mRNAs, miRNAs, and circRNAs for breast cancer and constructed a regulatory network. Then, to explore the key genes involved in the regulatory network, they established a PPI network and applied the MCODE algorithm; as a result of this analysis, LPCAT1, CBX2 and EGFR were identified as potential hub genes. Shi et al. [49] proposed an approach to identify driver genes by integrating mutation data, expression data, and gene networks and reported EGFR and PRKDC as potential driver genes for GBM.

In the next stage of our study, the somatic mutation status of selected genes for biomarker analysis was determined. In addition, a new prognostic scoring method has been developed that uses mRNA expression, methylation and mutation states of biomarkers simultaneously. Finally, we obtained statistically significant results for GNG11, CBX2, CDKN3, ARHGEF10, CLN8, SEC61G and PTDSS1 genes in the survival analysis. Previous studies found in the literature about these genes are summarized below.

G protein subunit gamma 11 (GNG11), a constituent of G-proteins, plays a vital role in the transmembrane signaling system. It has been described as a hub gene or a candidate biomarker in different cancer types. Hua et al. [50] reported in their study that GNG11 acts as a hub gene in lung adenocarcinoma. Moreover, Shi et al. [51] observed that GNG11 was downregulated in lung cancer, and low expression of GNG11 was associated with worse OS for female lung cancer patients who never smoked. Buttarelli et al. [52] generated a ten-gene signature, including the downregulated GNG11 gene, that predicts response to first-line chemotherapy in high-grade serous ovarian cancer patients. Furthermore, Jiang et al. [53] identified that high expression of GNG11 is related toa poor prognosis in ovarian cancer patients. According to Zhang et al. [54], GNG11 is downregulated in tumor tissue, and is the core gene in protein–protein interaction network analysis for triple-negative breast cancer. In addition, Xing et al. [55] stated that GNG11 is one of the eighteen key genes identified for the treatment of colorectal cancer. In line with other studies, according to our study, the GNG11 gene was downregulated and highly methylated in both breast and colon tumor tissues compared to normal tissues. Moreover, according to the prognostic scoring method we developed, GNG11 was associated with poor survival by presenting low scores in breast cancer and high scores in colorectal cancer.

Chromobox 2 (CBX2) is a polycomb repressor complex subunit, and some studies classified it as an oncogene. Clermont et al. [56] reported CBX2 as a potential drug target in their study and associated CBX2 expression with poor clinical outcomes in prostate cancer. Previous studies have shown that high expression of CBX2 is associated with worse survival in hepatocellular carcinoma, high-grade serous ovarian cancer, and lung adenocarcinoma [57,58,59]. Conversely, Ma et al. [60] identified that CBX2 mRNA and protein levels were significantly increased in gastric cancer tissues, but these levels were not significantly associated with the overall survival of patients. Furthermore, studies on colorectal cancer (CRC) showed that the CBX2 gene was significantly upregulated in CRC tissues compared to normal tissues, and this may be associated with poor prognosis [61,62]. There are various studies about the function of CBX2 in breast cancer. Bilton et al. [63] identified novel mechanisms by which CBX2 promotes breast cancer growth, and inhibition of CBX2 could be a novel therapeutic strategy. Zheng et al. [64] stated that there was a positive correlation between high CBX2 expression and activation of the PI3K/AKT pathway, and that CBX2 could be a potential prognostic biomarker. Li et al. [65] showed that the expression of CBX2 was strongly associated with tumor stage, and there was higher CBX2 expression in stage IV patients compared to others. Moreover, Piqué et al. [66] found that CBX2 promotes cell proliferation in breast cancer, its overexpression causes upregulation of genes involved in cell cycle progression, and CBX2 overexpression is associated with poor 5-year survival. Our results are consistent with previous studies; we found that CBX2 is upregulated in breast and clear-cell renal cell carcinoma and patients with poor survival showed higher prognostic scores in both cancer types.

Cyclin-dependent kinase 3 (CDKN3) is a member of the protein phosphatase inhibitors family and involved in regulation of the cell cycle [67,68]. Abnormal expression of CDKN3 is seen in many types of cancer. Abdel-Tawab et al. [69] suggested that CDKN3 expression could be used as a diagnostic and predictive biomarker of gastric cancer. Li et al. [70] found that CDKN3 was overexpressed in human gastric cancer tissues and associated with poor patient survival. Similarly, there are other studies associating CDKN3 overexpression with poor survival in nasopharyngeal carcinoma, lung adenocarcinoma, breast, bladder, and cervical cancer [71,72,73,74,75]. An immunohistochemical study for ESCC identified abnormal CDKN3 protein expression in esophageal squamous-cell cancer (ESCC) patients and confirmed its association with ESCC progression [76]. Yang and Sun [77] showed the role of CDKN3 in cellular proliferation of colorectal cancer by examining the effects of CDKN3 siRNA on the SW480 cell line; it is associated with cell cycle progression and apoptosis. Moreover, Li et al. [78] stated that CDKN3 is highly expressed in colorectal cancer, and this may be closely related to the poor prognosis of the patients. In our study using a different dataset, we found that the CDKN3 gene was highly expressed and less methylated in colorectal cancer patients compared to normal samples. In parallel with literature studies, we identified that colorectal cancer patients with poor survival showed a high prognostic score.

ARHGEF10 encodes the Rho guanine nucleotide exchange factor, and its role in cancer has not yet been clarified. However, there are studies presenting it as a candidate tumor suppressor gene for pancreatic ductal adenocarcinoma [79], hepatocellular carcinoma [80], breast [81] and urothelial carcinoma [82]. In addition, while decreased ARHGEF10 expression was observed in tumor cells in these studies, increased ARHGEF10 expression was found in colorectal cancer in our study. In addition, according to the prognostic scoring method we developed, high scores in colorectal cancer were associated with poor survival.

CLN8 encodes a transmembrane protein, and mutations in this gene are linked to progressive epilepsy with cognitive disabilities (EPMR), a subtype of neuronal ceroidlipofuscinoses (NCL) [83]. In order to reveal the role of NCL genes in cancer-related processes, Yap et al. [84] stated that the CLN8 gene showed low expression in brain cancer cells and had a tumor suppressor effect on patient survival. However, more research is needed in the future to explore the importance of CLN8 in cancers. In our study, low expression was observed in colorectal cancer tissue, and patients with high score values showed poor survival, according to the developed prognostic scoring method.

The subunit of the SEC61 translocon complex (SEC61G) participates in protein folding, post-translational modification and translocation, and plays critical roles in several cancer types [85]. Zhang et al. [86] used the expression levels of five genes to develop a prognostic model for colorectal cancer; one of these genes was SEC61G. In studies conducted for breast cancer, it has been stated that the SEC61G gene can be used as a diagnostic biomarker and therapeutic target, since high expression of SEC61G is associated with the expression of the proliferation marker Ki-67 and glycolysis. It was stated that SEC61G expression was higher and methylation level was lower in tumors compared to normal tissues, and this was associated with poor survival [87,88]. Zhang et al. [89] similarly found that SEC61G showed hypomethylation and high expression in bladder cancer cells. Meng et al. [90] stated that SEC61G is up-regulated in human kidney tumors and is associated with poor prognosis, compared with the control group. SEC61G knockdown significantly inhibits cell proliferation, migration and invasion; therefore it may serve as a biomarker for kidney cancer. In addition, some studies associated SEC61G overexpression with worse survival in hepatocellular carcinoma, head and neck squamous carcinoma, glioblastoma and lung adenocarcinoma [91,92,93,94]. In our study, we found that the SEC61G gene was highly expressed and hypo-methylated in lung cancer patients compared to normal samples. Furthermore, we identified that lung cancer patients with poor survival had high prognostic scores. Therefore, our analysis is supported by various literature studies.

There have been some cancer-related reports addressing phosphatidylserine synthase 1 (PTDSS1). Cheng et al. [95] stated that the PTDSS1 could be one of the anti-cancer targets for the treatment of colorectal cancer. Sekar et al. [96] showed that inhibiting the production of ether-phosphatidylserine by targeting PTDSS1 limits tumor-associated macrophage expansion and breast tumor growth. In a study on ESCC, it was stated that mRNA expression has a differential significance between ESCC and normal controls [97]. N’Guessan et al. [98] measured the expression of PTDSS1 at each stage of the cell cycle and found that PTDSS1 gene expression increased in the G2/M phase compared to the G1 phase in pancreatic cancer cells. They also noted that PTDSS1 gene expression was higher in pancreatic cancer patients compared to healthy tissues, and this was associated with a lower probability of survival in pancreatic cancer patients. In another research study, Li et al. [99] identified that high expression of PTDSS1 is significantly associated with a lower probability of survival in urothelial bladder carcinoma (BLCA), concluding that PTDSS1-mediated phosphatidylserine signaling is involved in the pathogenesis of BLCA. Furthermore, Wang et al. [100] concluded in their study that PTDSS1 is an oncogene in lung adenocarcinoma and its overexpression may reduce the likelihood of survival. In our study, we found that the PTDSS1 gene was upregulated and hypomethylated in LUSC compared to normal tissues. Moreover, according to the prognostic scoring method we developed, low scores in PTDSS1 were associated with poor survival.

In addition to carcinoma, potential biomarker genes in Table 13 have been associated with a wide range of other diseases, and they seem to be activated or inhibited in various biological processes. Cheng et al. [101] suggested that GNG11 could be used as a biomarker for differentiate ulcerative colitis and Crohn’s disease. Moradi et al. [102] proposed that GNG11 could be a diagnostic biomarker for Parkinson’s disease. GNG11 plays a key role in heart rhythm regulation and is associated with cardiac disease risk [103]. The CDKN3 gene could be used as a potential marker to identify severe COVID-19 patients [104]. Yue et al. [105] identified 10 central genes, including CDKN3, and stated that these genes may serve as new target markers for early diagnosis, prognosis and therapy in psoriasis. Yao et al. [106] constructed an index using seven genes, including CDKN3, which are associated with hypoxia, a prominent factor in the diagnosis and treatment of osteoarthritis. The ARHGEF10 mutation was associated with slowed nerve conduction velocity [107]. The ARHGEF10 gene might be associated with the pathogenesis of Behcet’s disease [108]. Zhang et al. [109] revealed the candidacy of the CLN8 gene as a genetic modifier contributing to extreme phenotypic variation in Gaucher disease. A novel mutation in CLN8 may cause Northern Epilepsy cases in Turkey [110]. CBX2 gene plays a role in the human sex development process and its disorders [111]. SEC61G is among the nine circadian-related genes identified related to circadian rhythm disruption, which is critical in the pathogenesis of Alzheimer’s disease [112]. The SEC61G gene was differently methylated in patients with Balkan endemic nephropathy [113]. The SEC61G gene is differentially expressed and methylated in fetal alcohol spectrum disorder patients [114]. There are many studies about relationship between the mutation in the PTDSS1 gene and Lenz-Majewski syndrome [115]. Soueid et al. [116] proposed that PTDSS1 is among the potential autism susceptibility genes in their study.

Besides the SNF method, MOFA has also been applied for the integration of gene expression, DNA methylation and mutation data. There are differences in the application of the two methods and the interpretation of the obtained results. The SNF method initially constructs a distinct similarity network for each omics, then integrates the networks using an iterative procedure. However, MOFA utilizes a matrix factorization technique. Although matrix factorization techniques are frequently employed for dimensionality reduction, they might ignore biological correlations between the features. Furthermore, because of its linearity, the MOFA model may miss non-linear correlations between features. Another challenging process was the biological interpretation of the inferred latent factors. Each feature in MOFA has a ‘weight’ that represents its relative relevance to the factor. We utilized these weights to assess the most informative biomarkers. The most difficult aspect of implementing the SNF method was integrating clinical data and was not included in this method. On the other hand, this capability is available in MOFA. Consequently, the two methodologies cannot be directly comparable in terms of their results due to their different computational methods. Despite all the differences of the two methods, they report similar gene outputs for some cancer phenotypes.

This study provides new insights into potential prognostic biomarkers for many tumor types; however, it has some limitations. First, gene expression, DNA methylation and mutation data of the same patient are required to calculate the proposed prognostic score. Nevertheless, in some cases, all three data types may not be available for the same patient. Second, all omics data come from the TCGA database; when other public repositories are checked, it is common to find gene expression data for specific drug treatments on cancer cell lines or gene knockout studies. In contrast, the samples in our study were selected from patients who did not receive any treatment. Furthermore, due to focusing on patients at cancer stage 1 and 2, there were relatively few samples remaining in the study. If a larger sample size is used, the predictive power of the algorithm can be more effectively verified. Although verification of the proposed biomarkers on a new patient cohort could not be currently applied, we aim to investigate the biological validity of some of these biomarkers in new patient cohorts as a future study.

## 5. Conclusions

We implemented an integrative network analysis approach that explores common biomarkers for lung, breast, colorectal and kidney cancers by integrating RNA-sequencing and DNA methylation data. Several network clustering algorithms were used on the integrated network data. Cancer-specific evaluation metrics were applied to evaluate clustering results, and finally, common modules were reported across four cancers. The same analysis pipeline was applied to the validation set and final prospective biomarkers were identified. Survival analysis for biomarkers was conducted with a new prognostic scoring method that integrates mRNA expression, methylation and mutation status of genes. A literature survey about significant biomarkers highlighted in survival analysis revealed that GNG11, CBX2, CDKN3, ARHGEF10, CLN8, SEC61G and PTDSS1 genes present similar survival and prognostic behaviors in the specified cancers. In summary, multi-omics and network-based analysis can help to discover new targets across cancers and to reduce treatment costs.

## Figures and Tables

**Table 1:** The number of cases by cancer types in the data set.

Cancer Type	Numberof Training Samples		Numberof Validation Samples	
	Tumor	Normal	Tumor	Normal
COAD	74	19	78	19
KIRC	90	24	91	24
BRCA	261	83	279	83
LUSC	153	7	152	7

**Table 2:** The numbers of common DEMG in the training and validation set. DEMG_Common indicates this number for each cancer type.

Cancer Type	DEMG_Common
Brca_hypo	2428
Lusc_hypo	3235
Coad_hypo	3382
Kırc_hypo	3184
Brca_hyper	2288
Lusc_hyper	1749
Coad_hyper	1475
Kırc_hyper	1063

**Table 3:** Summary of differential methylation analysis for the training set. “Hypo-M” and “Hyper-M” indicate hypomethylated and hypermethylated, respectively.

Cancer Type	Number of Differentially Methylated Probes		Number of Nearby Genes		Number of Probe-Gene Pairs	
	Hypo-M	Hyper-M	Hypo-M	Hyper-M	Hypo-M	Hyper-M
COAD	3103	2195	62,039	43,895	2561	6117
KIRC	1277	691	25,540	13,820	2388	2277
BRCA	1252	1048	25,040	20,953	2490	4606
LUSC	3415	1949	68,300	38,980	2588	3451

**Table 4:** Summary of differential methylation analysis for the validation set. “Hypo-M” and “Hyper-M” indicate hypomethylated and hypermethylated, respectively.

Cancer Type	Number of Differentially Methylated Probes		Number of Nearby Genes		Number of Probe-Gene Pairs	
	Hypo-M	Hyper-M	Hypo-M	Hyper-M	Hypo-M	Hyper-M
COAD	3084	1729	61,666	34,580	5324	3615
KIRC	1809	780	36,180	15,600	3440	2458
BRCA	1436	1278	28,720	18,180	2925	4225
LUSC	2121	1957	42,420	39,140	1543	4737

**Table 5:** Differential analysis results for RNA sequencing and methylation data in the training set.

Cancer Type	DEG	DMG_Hypo	DMG_Hyper	DEMG_Hypo	DEMG_Hyper
COAD	10,916	10,676	5012	4581	2211
KIRC	12,273	7005	2524	3556	1323
BRCA	14,294	4971	4773	2806	2812
LUSC	11,585	10,898	4666	5085	2309

DEG indicates differentially expressed gene numbers, DMG_Hypo indicates differentially hypomethylated gene numbers, DMG_Hyper indicates differentially hypermethylated gene numbers, DEMG_Hypo indicates differentially expressed and hypomethylated gene numbers, and DEMG_Hyper indicates differentially expressed and hypermethylated gene numbers.

**Table 6:** Differential analysis results for RNA sequencing and methylation data in the validation set.

Cancer Type	DEG	DMG_Hypo	DMG_Hyper	DEMG_Hypo	DEMG_Hyper
COAD	11,815	10,426	4417	4886	2095
KIRC	14,087	9177	2655	5325	1578
BRCA	14,667	5510	4547	3228	2747
LUSC	12,147	8442	4733	4040	2412

DEG indicates differentially expressed gene numbers, DMG_Hypo indicates differentially hypomethylated gene numbers, DMG_Hyper indicates differentially hypermethylated gene numbers, DEMG_Hypo indicates differentially expressed and hypomethylated gene numbers, and DEMG_Hyper indicates differentially expressed and hypermethylated gene numbers.

**Table 7:** Performance comparison of clustering algorithms between DEMG-Hyper data for the training set.

	Average-Bioscore (GO-BP)	Average-Bioscore (KEGG)	Average- BHI	# of Cluster
BRCA_hyper				
Fast Greedy	0.500	0.596	0.077	27
Infomap	0.229	0.144	0.055	257
Louvin	0.400	0.422	0.069	30
COAD_hyper				
Fast Greedy	0.289	0.427	0.069	20
Infomap	0.178	0.128	0.046	227
Louvin	0.358	0.328	0.065	27
KIRC_hyper				
Fast Greedy	0.449	0.126	0.067	15
Infomap	0.164	0.007	0.044	144
Louvin	0.342	0.05	0.056	20
LUSC_hyper				
Fast Greedy	0.409	0.446	0.072	24
Infomap	0.135	0.032	0.049	213
Louvin	0.304	0.217	0.065	31

**Table 8:** Performance comparison of clustering algorithms between DEMG-Hypo data for the training set.

	Average-Bioscore (GO-BP)	Average-Bioscore (KEGG)	Average- BHI	# of Cluster
BRCA_hypo				
Fast Greedy	0.427	0.539	0.081	21
Infomap	0.117	0.094	0.042	274
Louvin	0.484	0.465	0.071	30
COAD_hypo				
Fast Greedy	0.516	0.453	0.082	19
Infomap	0.132	0.064	0.042	434
Louvin	0.467	0.374	0.08	35
KIRC_hypo				
Fast Greedy	0.525	0.499	0.083	18
Infomap	0.176	0.112	0.04	377
Louvin	0.387	0.472	0.071	32
LUSC_hypo				
Fast Greedy	0.274	0.517	0.08	25
Infomap	0.08	0.026	0.043	393
Louvin	0.525	0.351	0.074	37

**Table 9:** Performance comparison of clustering algorithms between DEMG-Hyper data for the validation set.

	Average-Bioscore (GO-BP)	Average-Bioscore (KEGG)	Average- BHI	# of Cluster
BRCA_hyper				
Fast Greedy	0.515	0.371	0.074	25
Infomap	0.187	0.096	0.066	246
Louvin	0.253	0.476	0.044	32
COAD_hyper				
Fast Greedy	0.512	0.105	0.062	17
Infomap	0.246	0.011	0.064	194
Louvin	0.359	0.057	0.050	27
KIRC_hyper				
Fast Greedy	0.346	0.186	0.077	14
Infomap	0.191	0.106	0.071	173
Louvin	0.361	0.383	0.048	22
LUSC_hyper				
Fast Greedy	0.460	0.349	0.074	23
Infomap	0.147	0.081	0.075	220
Louvin	0.543	0.379	0.050	30

**Table 10:** Performance comparison of clustering algorithms between DEMG-Hypo data for the validation set.

	Average-Bioscore (GO-BP)	Average-Bioscore (KEGG)	Average- BHI	# of Cluster
BRCA_hypo				
Fast Greedy	0.526	0.294	0.076	23
Infomap	0.045	0.025	0.051	295
Louvin	0.299	0.243	0.077	31
COAD_hypo				
Fast Greedy	0.305	0.567	0.089	20
Infomap	0.193	0.134	0.083	424
Louvin	0.487	0.545	0.056	29
KIRC_hypo				
Fast Greedy	0.459	0.454	0.080	21
Infomap	0.170	0.056	0.039	525
Louvin	0.415	0.199	0.074	32
LUSC_hypo				
Fast Greedy	0.322	0.229	0.076	25
Infomap	0.087	0.036	0.070	336
Louvin	0.415	0.287	0.055	33

**Table 11:** Common genes in the same module for both training and validation sets.

Genes Name	Methylation Group
PRKDC, MCM4, UBE2V2	Hypo-methylated
LPCAT1, mrpl36	Hypo-methylated
CDKN3, CGRRF1	Hypo-methylated
GNG11, GNGT1	Hypo-methylated
ACTR3B, IMMP2L	Hypo-methylated
SEC61G, EGFR	Hypo-methylated
PTDSS1, CPQ	Hypo-methylated
ARHGEF10, CLN8	Hypo-methylated
CBX8, CBX2	Hypo-methylated
RAN, ADGRD1	Hypo-methylated
TPRG1L, PRDM16-DT	Hypo-methylated
LGR4, BDNF-AS	Hypo-methylated
SLC9A3, PP7080	Hyper-methylated
ENPP5, CYP39A1	Hyper-methylated
RAD54L, EFCAB14	Hyper-methylated
BRIP1, TBX2-AS1	Hyper-methylated

**Table 12:** Average cutoff values for the high and low levels.

Cancer Type	Average of Low-Level Scores	Average of High-Level Scores
Brca_hypo	0.277	0.419
Lusc_hypo	0.282	0.41
Coad_hypo	0.279	0.437
Kırc_hypo	0.276	0.381
Brca_hyper	0.314	0.457
Lusc_hyper	0.285	0.434
Coad_hyper	0.324	0.49
Kırc_hyper	0.364	0.489

**Table 13:** Survival analysis Cox PH model results.

Cancer Type	Gene Name	Prognostic Score Level	Hazard Rate	*p*-Value	Number of Patients at Score Level	Number of Deaths
Brca_hypo	GNG11	Low	7.7055	0.000189	11	4
	CBX2	High	2.0370	0.0138	188	27
Coad_hypo	CDKN3	High	2.577	0.0262	64	15
	ARHGEF10	High	2.855	0.0128	56	14
	GNG11	High	2.2279	0.0563	45	12
	CLN8	High	3.037	0.00823	53	14
Kırc_hypo	CBX2	High	2.8296	0.02	19	7
Lusc_hypo	SEC61G	High	1.6608	0.0541	239	99
	PTDSS1	High	2.6287	0.0217	273	111

## Data Availability

All patient samples are available from the GDC data portal https://portal.gdc.cancer.gov (accessed on 20 February 2022).

## Supplementary Materials

The following supporting information can be downloaded at: https://www.mdpi.com/article/10.3390/medsci11030044/s1, File S1: Basic clinical characteristics of patients; File S2: Kaplan–Meier plots of potential biomarker genes; File S3: The results of survival analysis for the weights with gene expression (0.4), DNA methylation (0.4), and mutation (0.2); File S4: The results of survival analysis with individual data types; File S5: The results of MOFA analysis; Table S1: Cancer-related terms used in Bioscore for GO BP; Table S2: Cancer-related terms used in Bioscore for KEGG pathway.
