# Supplementary material for: Multi-Omics Data Analysis Identifies Prognostic Biomarkers across Cancers
Source: Med Sci (Basel). 2023 Jun 27;11(3):44. doi: 10.3390/medsci11030044 (PMC10366886; doi:10.3390/medsci11030044)
Supplement: Supplementary file 1 [file medsci-11-00044-s001.zip › File S1.pdf]

Baseline clinical characteristics of the TCGA - BRCA training set and validation set

BRCA	Training set		Validation set		
Characteristics	Number of cases	%	Number of cases	%	Chi-square test *p*-value
**Age**					
<60	132	50.6	155	55.6	0.2834
≥60	129	49.4	124	44.4	
**Gender**					
Female	258	98.8	277	99.3	0.9403
Male	3	1.2	2	0.7	
**Status**					
Alive	231	88.5	255	91.4	0.3291
Dead	30	11.5	24	8.6	
**Stage**					
Stage I	61	23.4	62	22.2	0.8293
Stage II	200	76.6	217	77.8	

Baseline clinical characteristics of the TCGA - LUSC training set and validation set

LUSC	Training set		Validation set		
Characteristics	Number of cases	%	Number of cases	%	Chi-square test *p*-value
**Age**					
<60	21	13.7	30	19.7	0.2101
≥60	132	86.3	122	80.3	
**Gender**					
Female	50	32.7	32	21	0.03071
Male	103	67.3	120	79	
**Status**					
Alive	79	51.6	106	69.7	0.001817
Dead	74	48.4	46	30.3	
**Stage**					
Stage I	86	56.2	86	56.6	1
Stage II	67	43.8	66	43.4	

Baseline clinical characteristics of the TCGA - COAD training set and validation set

**COAD**	**Training set**		**Validation set**		
Characteristics	Number of cases	%	Number of cases	%	Chi-square test *p*-value
**Age**					
<60	21	28.4	20	25.6	0.8436
≥60	53	71.6	58	74.4	
**Gender**					
Female	36	48.6	36	46.2	0.8844
Male	38	51.4	42	53.8	
**Status**					
Alive	64	86.5	64	82	0.5982
Dead	10	13.5	14	18	
**Stage**					
Stage I	22	29.7	22	28.2	0.9775
Stage II	52	70.3	56	71.8	

Baseline clinical characteristics of the TCGA - KIRC training set and validation set

**KIRC**	**Training set**		**Validation set**		
Characteristics	Number of cases	%	Number of cases	%	Chi-square test *p*-value
**Age**					
<60	45	50	46	50.5	1
≥60	45	50	45	49.5	
**Gender**					
Female	40	44.4	33	36.3	0.3319
Male	50	55.6	58	63.7	
**Status**					
Alive	76	84.4	77	84.6	1
Dead	14	15.6	14	15.4	
**Stage**					
Stage I	81	90	76	83.5	0.2861
Stage II	9	10	15	16.5	
