# Supplementary material for: Multi-Omics Data Analysis Identifies Prognostic Biomarkers across Cancers
Source: Med Sci (Basel). 2023 Jun 27;11(3):44. doi: 10.3390/medsci11030044 (PMC10366886; doi:10.3390/medsci11030044)
Supplement: Supplementary file 1 [file medsci-11-00044-s001.zip › File S2.pdf]

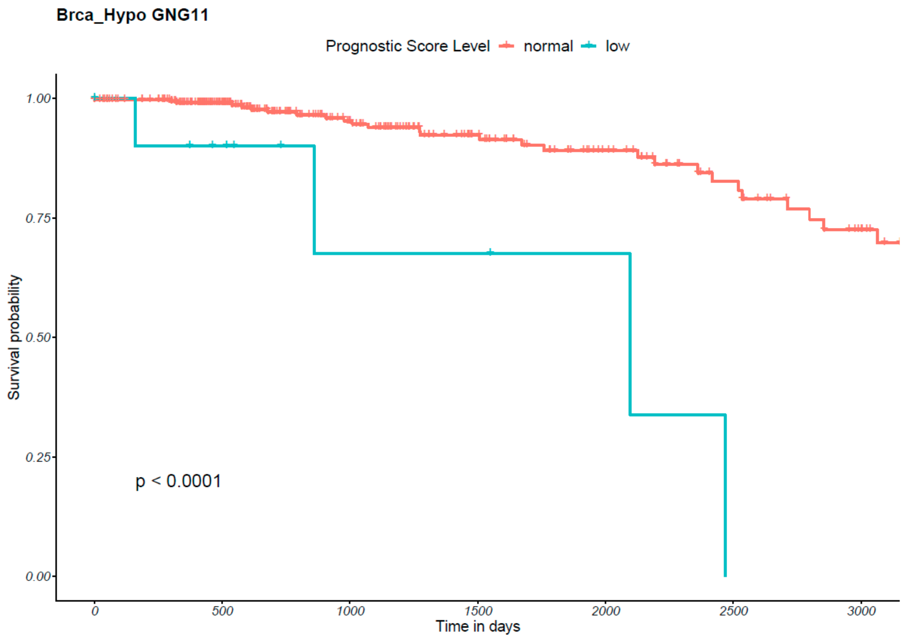

Kaplan Meier analysis result for the Brca_Hypo GNG11 gene

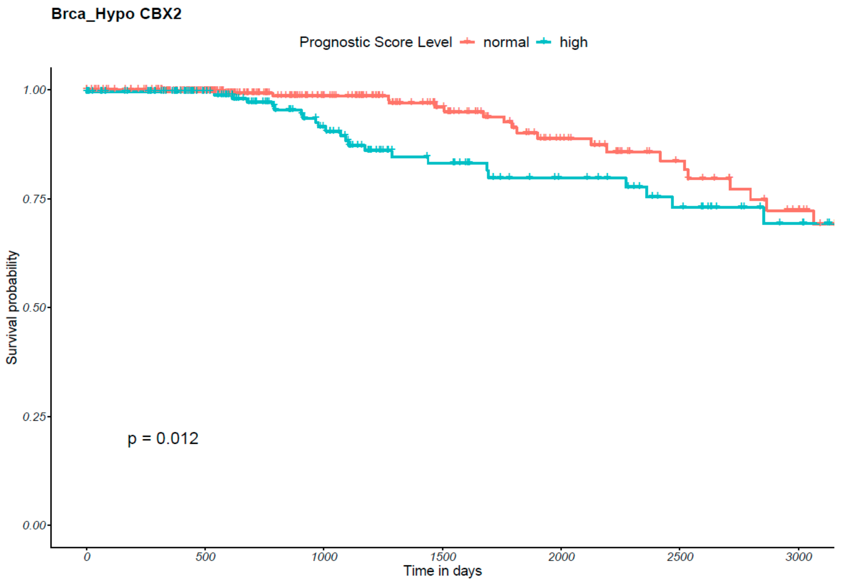

Kaplan Meier analysis result for the Brca_Hypo CBX2 gene

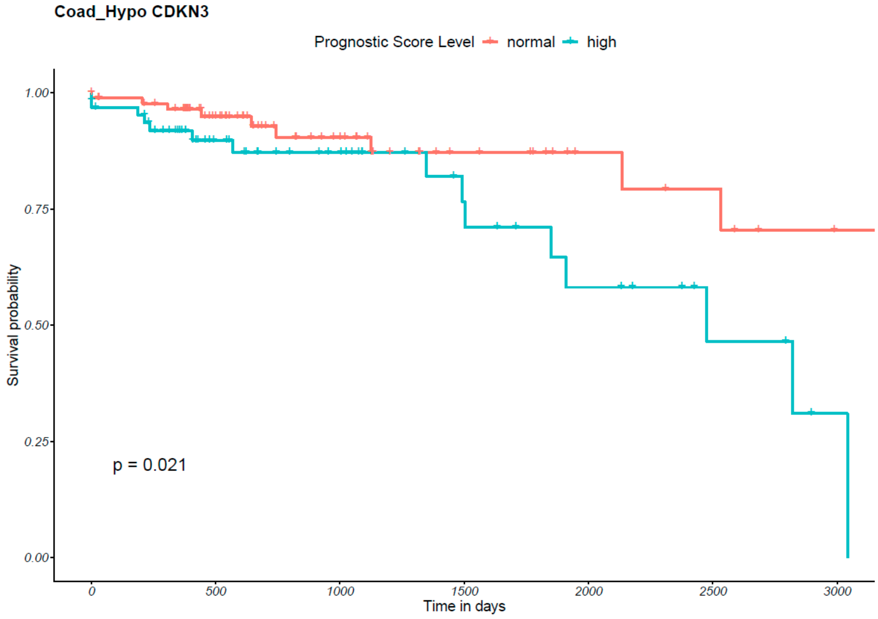

Kaplan Meier analysis result for the Coad_Hypo CDKN3 gene

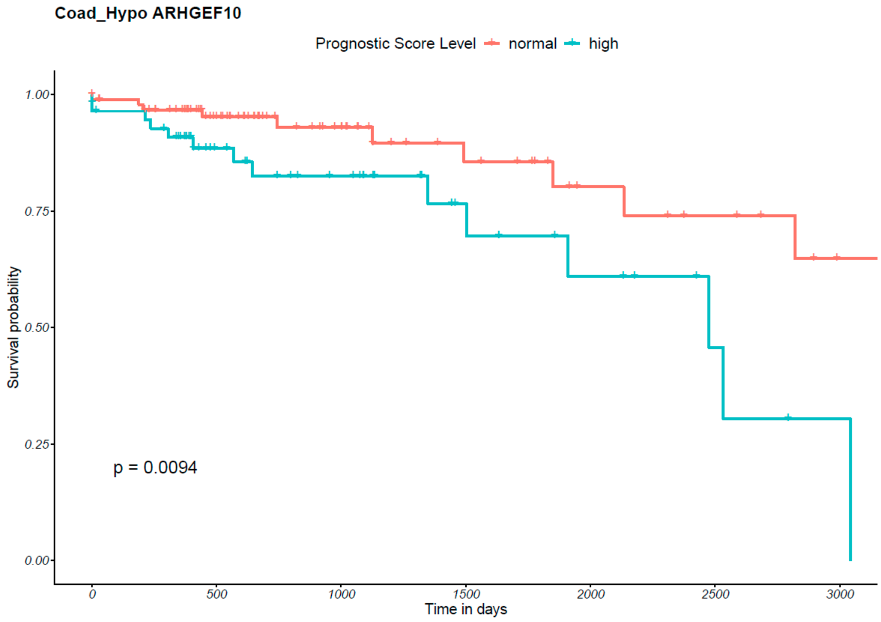

Kaplan Meier analysis result for the Coad _Hypo ARHGEF10 gene

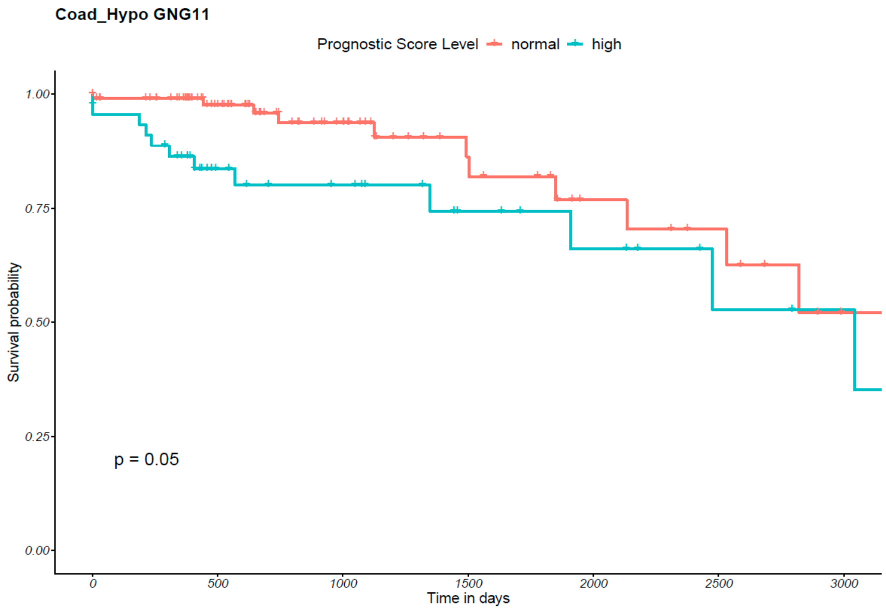

Kaplan Meier analysis result for the Coad _Hypo GNG11 gene

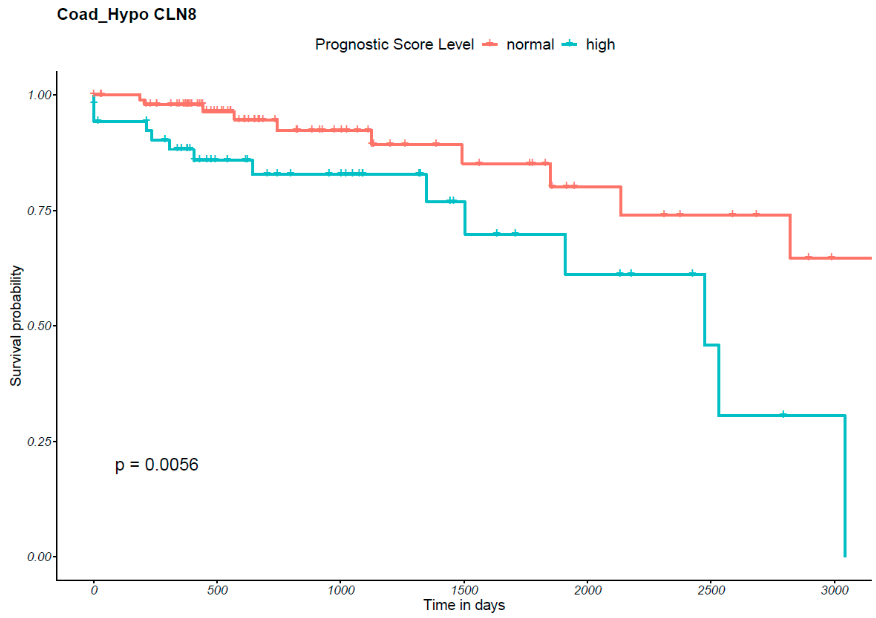

Kaplan Meier analysis result for the Coad _Hypo CLN8 gene

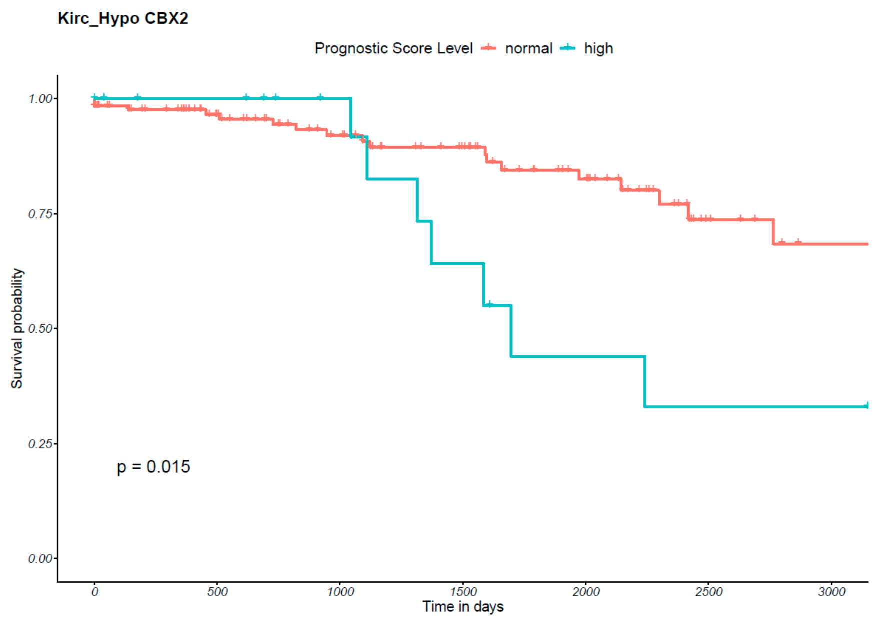

Kaplan Meier analysis result for the Kırc_Hypo CBX2 gene

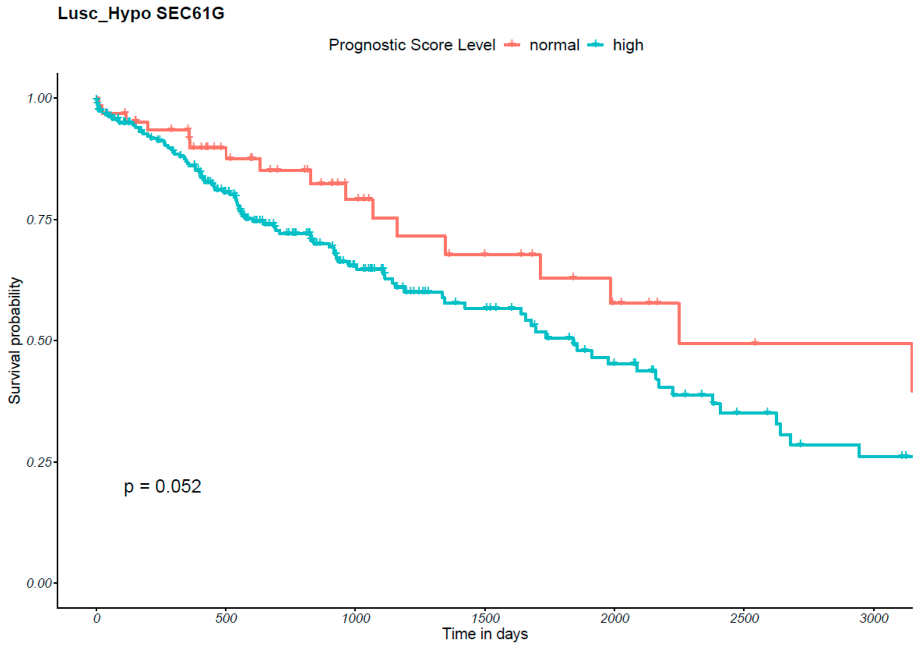

Kaplan Meier analysis result for the Lusc_Hypo SEC61G gene

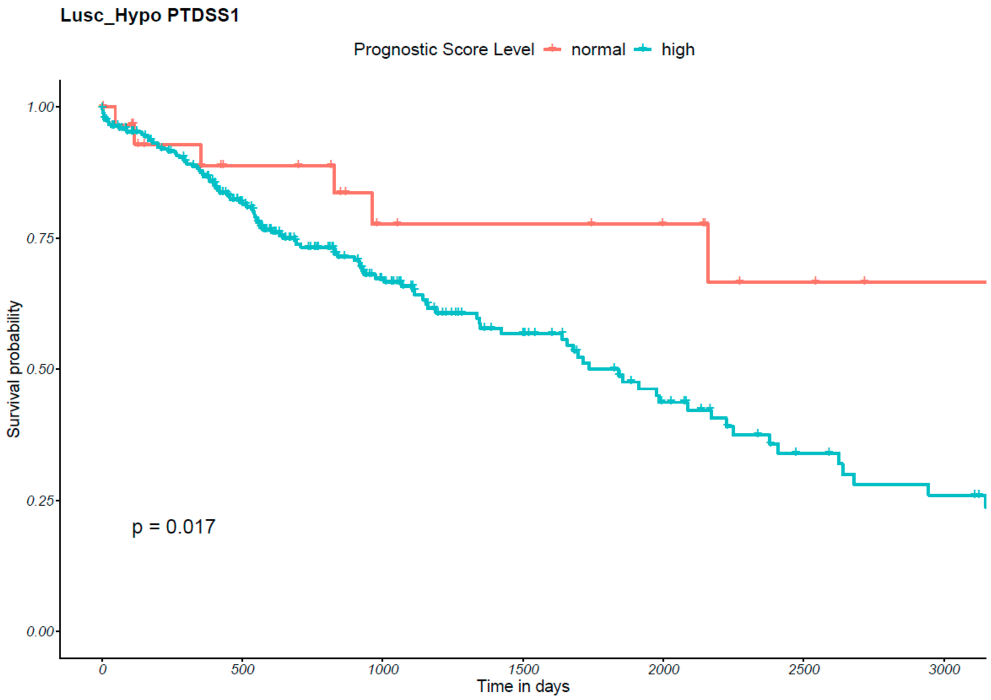

Kaplan Meier analysis result for the Lusc_Hypo PTDSS1 gene
