# Supplementary material for: Multi-Omics Data Analysis Identifies Prognostic Biomarkers across Cancers
Source: Med Sci (Basel). 2023 Jun 27;11(3):44. doi: 10.3390/medsci11030044 (PMC10366886; doi:10.3390/medsci11030044)
Supplement: Supplementary file 1 [file medsci-11-00044-s001.zip › File S3.pdf]

Average cut-off values for the high and low levels. (0.4 – 0.4 – 0.2 version)

Cancer Type	Average of Low-Level Scores	Average of High-Level Scores
**Brca_hypo**	0.264	0.437
**Lusc_hypo**	0.275	0.424
**Coad_hypo**	0.264	0.438
**Kırc_hypo**	0.253	0.379
**Brca_hyper**	0.312	0.485
**Lusc_hyper**	0.283	0.454
**Coad_hyper**	0.323	0.508
**Kırc_hyper**	0.371	0.524

Survival analysis Cox PH model results (0.4 – 0.4 – 0.2 version)

Cancer Type	Gene Name	Prognostic Score Level	Hazard Rate	P-value	Number of patients at score level	Number of deaths
**Brca_hypo**	PRDM16-DT	high	3.8503	0.00233	43	7
**Coad_hypo**	LPCAT1	low	5.5965	0.00109	47	12
**Lusc_hypo**	BDNF-AS	high	1.5783	0.04650	62	29
	IMMP2L	high	2.6882	0.00127	28	15
