# Supplementary material for: Multi-Omics Data Analysis Identifies Prognostic Biomarkers across Cancers
Source: Med Sci (Basel). 2023 Jun 27;11(3):44. doi: 10.3390/medsci11030044 (PMC10366886; doi:10.3390/medsci11030044)
Supplement: Supplementary file 1 [file medsci-11-00044-s001.zip › File S4.pdf]

Survival analysis result using only gene expression

Cancer Type	Average of Low-Level Scores	Average of High-Level Scores
**Brca_hypo**	**0.36**	**0.494**
**Lusc_hypo**	**0.347**	**0.487**
**Coad_hypo**	**0.374**	**0.563**
**Kırc_hypo**	**0.401**	**0.515**
**Brca_hyper**	**0.365**	**0.497**
**Lusc_hyper**	**0.335**	**0.4819**
**Coad_hyper**	**0.374**	**0.567**
**Kırc_hyper**	**0.394**	**0.509**

Cancer Type	Gene Name	Prognostic Score Level	Hazard Rate	P-value	Number of patients at score level	Number of deaths
**Lusc_hypo**	ADGRD1	low	1.6715	0.0129	102	46

Survival analysis result using only DNA methylation

Cancer Type	Average of Low-Level Scores	Average of High-Level Scores
**Brca_hypo**	**0.247**	**0.648**
**Lusc_hypo**	**0.287**	**0.618**
**Coad_hypo**	**0.224**	**0.567**
**Kırc_hypo**	**0.187**	**0.476**
**Brca_hyper**	**0.364**	**0.765**
**Lusc_hyper**	**0.325**	**0.700**
**Coad_hyper**	**0.362**	**0.750**
**Kırc_hyper**	**0.488**	**0.846**

Cancer Type	Gene Name	Prognostic Score Level	Hazard Rate	P-value	Number of patients at score level	Number of deaths
**Coad_hypo**	CPQ	High	3.280e-01	0.0456	44	4
**Lusc_hypo**	LPCAT1	high	2.1294	0.00439	40	20
	IMMP2L	low	1.7847	0.032	44	19
	IMMP2L	high	1.7056	0.0112	97	42

Survival analysis result using only mutation status

Cancer Type	Gene Name	Hazard Rate	P-value	Number of patients with the mutation	Number of deaths
**Brca_hypo**	ADGRD1	41.302	0.001	5	1
**Coad_hyper**	SLC9A3	3.791	0.009	9	5
	BRIP1	3.096	0.069	7	3
