# Supplementary material for: Multi-Omics Data Analysis Identifies Prognostic Biomarkers across Cancers
Source: Med Sci (Basel). 2023 Jun 27;11(3):44. doi: 10.3390/medsci11030044 (PMC10366886; doi:10.3390/medsci11030044)
Supplement: Supplementary file 1 [file medsci-11-00044-s001.zip › File S5.pdf]

Survival analysis Cox PH model results for MOFA factors. (Results with hazard ratio > 1.0 and *p*-value < 0.05 are presented.)

Cancer Type	Factor Name	Hazard Rate	P-value
Brca_hypo	Factor 7	1.7714	0.0001
	Factor 15	1.5109	0.0198
Coad_hypo	Factor 7	2.053	0.0201
Kırc_hypo	Factor 8	4.5468	0.0005
Brca_hyper	Factor 4	1.3716	0.04
	Factor 8	1.4244	0.015

Similarities between the genes in significant MOFA factors and the genes in Tables 11 and 13 of the manuscript. Significant MOFA factors are identified as the first three factors through variance decomposition analysis, and the factors found to be significant in the survival analysis.

Cancer Type	Gene Name	Factor Name	Omics Data Type
Brca_hypo	EGFR	Factor 2	Mutation
	PRKDC	Factor 2	Mutation
	EGFR	Factor 3	Mutation
	PRKDC	Factor 3	Mutation
	CBX2	Factor 7	Gene expression
	ADGRD1	Factor 7	Gene expression
	PRKDC	Factor 7	Mutation
	GNG11	Factor 15	Gene expression
Coad_hypo	PRKDC	Factor 1	Mutation
	TPRG1L	Factor 2	DNA Methylation
	LGR4	Factor 3	DNA Methylation
	BRIP1	Factor 3	DNA Methylation
	CDKN3	Factor 7	DNA Methylation
Kırc_hypo	MRPL36	Factor 2	DNA Methylation
	CLN8	Factor 2	Mutation
	RAN	Factor 3	DNA Methylation
	PRKDC	Factor 3	Mutation
	PRKDC	Factor 8	Mutation
	ADGRD1	Factor 8	Mutation
Lusc_hypo	LPCAT1	Factor 1	Gene expression
Brca_hyper	PRKDC	Factor 1	Mutation
	PRKDC	Factor 3	Mutation
	PRKDC	Factor 4	Mutation
	TBX2-AS1	Factor 8	Gene expression
Coad_hyper	SLC9A3	Factor 2	Mutation
	PRDM16-DT	Factor 3	DNA Methylation
Lusc_hyper	PRKDC	Factor 1	Mutation
	ENPP5	Factor 3	Gene expression
Kırc_hyper	PRDM16-DT	Factor 1	Gene expression
	CLN8	Factor 1	Mutation
	SLC9A3	Factor 2	Gene expression
	ENPP5	Factor 2	Gene expression
	CYP39A1	Factor 3	DNA Methylation
	ENPP5	Factor 3	DNA Methylation
	CLN8	Factor 3	Mutation
	BRIP1	Factor 3	Mutation
